# Design and implementation of a head‐and‐neck phantom for system audit and verification of intensity‐modulated radiation therapy

**DOI:** 10.1120/jacmp.v9i2.2740

**Published:** 2008-04-16

**Authors:** Gareth J. Webster, Mark J. Hardy, Carl G. Rowbottom, Ranald I. Mackay

**Affiliations:** ^1^ North Western Medical Physics Christie Hospital NHS Foundation Trust Manchester U.K.

**Keywords:** IMRT, verification, audit

## Abstract

The head and neck is a challenging anatomic site for intensity‐modulated radiation therapy (IMRT), requiring thorough testing of planning and treatment delivery systems. Ideally, the phantoms used should be anatomically realistic, have radiologic properties identical to those of the tissues concerned, and allow for the use of a variety of devices to verify dose and dose distribution in any target or normal‐tissue structure.

A phantom that approaches the foregoing characteristics has been designed and built; its specific purpose is verification for IMRT treatments in the head‐and‐neck region. This semi‐anatomic phantom, HANK, is constructed of Perspex (Imperial Chemical Industries, London, U.K.) and provides for the insertion of heterogeneities simulating air cavities in a range of fixed positions. Chamber inserts are manufactured to incorporate either a standard thimble ionization chamber (0.125 cm^3^: PTW, Freiburg, Germany) or a smaller PinPoint chamber (0.015 cm^3^: PTW), and measurements can be made with either chamber in a range of positions throughout the phantom. Coronal films can also be acquired within the phantom, and additional solid blocks of Perspex allow for transverse films to be acquired within the head region.

Initial studies using simple conventional head‐and‐neck plans established the reproducibility of the phantom and the measurement devices to within the setup uncertainty of ±0.5 mm. Subsequent verification of 9 clinical head‐and‐neck IMRT plans demonstrated the efficacy of the phantom in making a range of patient‐specific dose measurements in regions of dosimetric and clinical interest. Agreement between measured values and those predicted by the Pinnacle^3^ treatment planning system (Philips Medical Systems, Andover, MA) was found to be generally good, with a mean error on the calculated dose to each point of +0.2% (range: −4.3% to +2.2%;n=9) for the primary planning target volume (PTV), −0.1% (range: −1.5% to +2.0%;n=8) for the nodal PTV, and +0.0% (range: −1.8% to +4.3%;n=9) for the spinal cord. The suitability of the phantom for measuring combined dose distributions using radiographic film was also evaluated.

The phantom has proved to be a valuable tool in the development and implementation of clinical head‐and‐neck IMRT, allowing for accurate verification of absolute dose and dose distributions in regions of clinical and dosimetric interest.

PACS numbers: 87.53.‐j, 87.53.Xd, 87.56.Fc

## I. INTRODUCTION

The head and neck is a challenging site for treatment with intensity‐modulated radiotherapy (IMRT), and thorough testing of planning techniques and treatment delivery systems is required for safe introduction of that technique into clinical practice.^(^ ^1^ ^,^ ^2^ ^)^ The improved conformity of the dose delivered with IMRT to target structures and the sharp gradients between target and critical tissues can allow for dose escalation to the treatment volume while acceptable levels of radiation damage are maintained. The result can be increased proximity of high‐dose regions to critical structures.

The potential for a large dosimetric error between planned and delivered treatments means that several stages in the treatment process must be performed to a higher level of accuracy than is typically conventionally achieved. For example, the beam modeling by the treatment planning system (TPS) is likely to require improved accuracy as compared with the accuracy acceptable for conventional treatments, in which penumbral and out‐of‐field doses are less significant to the evaluation of the dose distribution. This issue arises because the contribution of out‐of‐field doses to clinically significant areas is much higher for IMRT than for conventional radiotherapy. For critical structures such as the spinal cord and brainstem, errors in such contributions to the calculated dose could misleadingly overestimate or, critically, underestimate the delivered dose to a sensitive tissue. Direct measurement in such regions can confirm the accuracy of the planned dose.

To provide an accurate representation of the patient contour, verification of IMRT techniques at both the commissioning and clinical stages should use phantoms that can provide a realistic representation of the clinical site being treated. Ideally, these phantoms should be anatomically realistic, have radiologic properties that are identical to those of the tissues concerned, and allow for a variety of measuring devices to be used to verify dose and dose distribution in a number of key positions throughout the target and normal‐tissue volumes. In addition, given the many links in the radiotherapy chain, it is important to minimize systematic errors in the process from imaging through treatment planning to the fractionated delivery of the radiation.^(1)^ A phantom that can test the various components of the system before clinical implementation of IMRT is vital to ensure confidence in the new technique.

To meet those requirements, a semi‐anatomic head‐and‐neck phantom was designed and manufactured at the Christie Hospital. The phantom can be used to verify clinical IMRT treatments in the head‐and‐neck region and to ensure that systematic errors in the process are minimized. The present paper details the considerations involved in the design of the phantom and the current successes and limitations resulting from its use.

## II. MATERIALS AND METHODS

**Figure 1:**
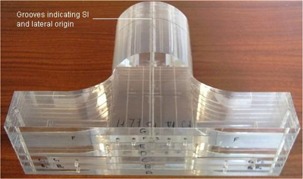
Semi‐anatomic Perspex (Imperial Chemical Industries, London, U.K.) phantom for verification of head‐and‐neck treatment delivery (HANK).SI=superior–inferior.

Table 1 summarizes the design considerations for the head‐and‐neck phantom and the features incorporated into the design to address those considerations. The approximately anatomically realistic shape of the phantom comprises a head section that links to a shoulder section (Fig. 1); together, the two parts approximate the contours of a typical patient. The choice of Perspex (Imperial Chemical Industries, London, U.K.) as the phantom material was made because of considerations of ease of availability and the similarity of that material's density to the average of tissue and bone in the human head. The validity of the density assumption was checked by comparing the average density of the phantom, including oral cavity and esophageal heterogeneities, to the average density of 7 head‐and‐neck IMRT patients from the lung apex to approximately the superior border of the spinal cord. The mean patient density was found to be $1.073\;g/{cm}^{3}$ (range: $1.018-1.236\;g/{cm}^{3}$) as compared with $1.076\pm 0.003\;g/{cm}^{3}$ for the phantom. The alternative option of manufacturing the phantom using a water‐equivalent material that allowed for the insertion of a comprehensive range of bone‐ and air‐equivalent heterogeneities, while being more anatomically realistic, was felt to compromise the simplicity and flexibility of the phantom. Given those limitations, the use of a material of mean tissue density was felt to provide a more practical patient representation. The solution developed here allows for an assessment of dose calculation accuracy at depths and obliquities similar to those for a patient.

**Figure 2:**
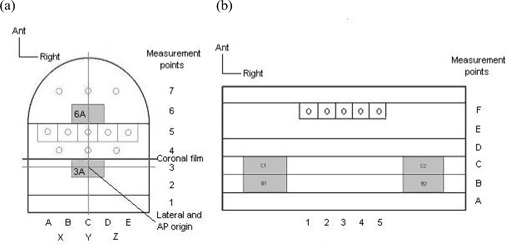
(a) Head section: The blocks containing slices 2 and 3, and 4 and 5 can be interchanged. (b) Shoulder section: Measurement points can be placed in slices C to F. The tracheal heterogeneity can be positioned at the center of the measurement slice. Blocks B and C are removable blocks simulating lung heterogeneities. Ant=anterior; AP=anterior−posterior.

**Figure 3:**
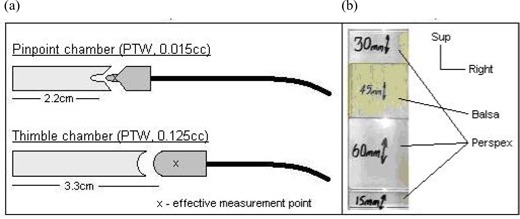
(a) Schematic of chamber inserts for the thimble (0.125 cm^3^: PTW, Freiburg, Germany) and PinPoint (0.015 cm^3^: PTW) ionization chambers. (b) Repeatable setup of the balsawood heterogeneity representing the oral cavity, viewed in the coronal plane of heterogeneity [see Figs. 2 and 6(a)]. Sup=superior.

Fig. 2 shows the structure of the head section of the phantom: a range of movable slabs and removable blocks that run craniocaudally through the head section. One of the blocks includes a cylindrical insert that can be removed to allow insertion of an ionization chamber for absolute dose measurement. Chamber inserts were designed with scalloped ends to tightly fit a thimble ionization chamber (0.125 cm^3^: PTW, Freiburg, Germany) and a PinPoint chamber (0.015 cm^3^: PTW), as illustrated in Fig. 3(a). Flat‐ended spacers are used as necessary to alter the superior—inferior position of the chamber for measurements. The shoulder section is fitted with a similar system of versatile measurement points, so that a three‐dimensional (3D) grid of 156 measurement points is available throughout the head and neck region. The relevant point can be selected at positions of clinical or dosimetric interest.

The ability to incorporate simple heterogeneities into the phantom not only permits a more realistic representation of a patient, but also allows for verification of heterogeneity correction in the TPS. Some heterogeneities can be simulated simply by removing existing inserts, for example in the lung (Fig. 2). However, it is often more anatomically realistic for an air cavity to be of a more limited length—for example, the oral cavity, which requires the air cavity to be of a known and repeatable length and position. Inserts have therefore been made to simulate typical heterogeneities. A balsa wood insert can replace a Perspex insert in the shoulder section of the phantom to simulate the trachea. Blocks of Perspex and balsa have also been made to simulate the oral cavity in the configuration shown in Fig. 3(b), which is positioned in the coronal plane in phantom section 6A [see Fig. 2(a)]. Balsa wood is a good material for this purpose because it has a low electron density, close to that of air $\left(0.07\;g/{cm}^{3}\right)$. The balsa wood inserts allow for repeatable positioning of the Perspex blocks for precise measurements near to the inhomogeneity.

**Table 1:** Design considerations for a verification phantom for intensity‐modulated radiation therapy of head and neck

*Design requirement*	*Solution*
Approximate patient shape	Separate sections were made for the head and the shoulders (Fig. 1) to generate a realistic surface contour.
Flexible design	Movement of various components is accommodated throughout the phantom's volume.
Stable geometric phantom with repeatable setup	The required flexibility of measurement positions limits the complexity of the phantom, and so a simple geometric design is used.
Radiologically realistic material	The phantom was manufactured from Perspex (Imperial Chemical Industries, London, U.K.), a material with a density comparable to that found to be the average of tissue and bone in the head‐and‐neck region.
An array of repeatable dose measurement points throughout the phantom volume	Both sections of the phantom have a slab structure (Fig. 2; head: two slabs; shoulder: one slab) containing movable blocks. The blocks, one of which can contain an ionization chamber, can be moved laterally, and the slabs can be interchanged. Spacers provide the superior—inferior flexibility needed to accurately position the ionization chamber.
Ability to use standard thimble ionization chambers (0.125 cm^3^: PTW, Freiburg, Germany) and PinPoint chambers (0.015 cm^3^: PTW)	Various chamber inserts were manufactured to securely hold both chamber types (Fig. 3).
Facility to include clinically relevant heterogeneities	Removable lung sections are included inferiorly. Balsawood inserts of density $0.07\;g/{cm}^{3}$ [Fig. 3(b)] simulate the oral cavity and trachea. For flexibility, other materials and different positions can be used.
Facility to acquire coronal films of combined dose distributions	Films can be positioned between certain slabs extending through both the head and the shoulder section of the phantom.
Facility to acquire transverse films of combined dose distributions	A separate phantom consisting of two Perspex slabs with surface contours identical to those of the head section of the original phantom was made (Fig. 4).

**Figure 4:**
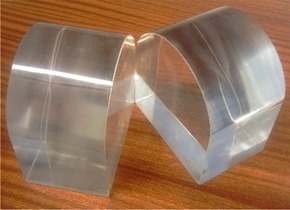
Head phantom for transverse film measurements.

The structure of the phantom permits radiographic film to be inserted between slabs 3 and 4 in the head, extending into the shoulder section if necessary, to measure the dose distribution in the coronal plane [see Fig. 2(a)]. No such capability exists for measuring transverse slices, because the phantom would then have to be split into several parts, rather than the current two, which would make the phantom impractical to use and would reduce its stability. A second phantom was therefore designed (Fig. 4). This simplified replica of the head section contains no heterogeneities or slab structure (the latter being unnecessary when measuring transverse slices). To measure the dose distribution of any transverse plane, a film can be placed between the slabs before the phantom is positioned and the treatment delivered.

Head‐and‐neck phantoms for radiotherapy have been developed elsewhere. Examples include the Rando phantom (originally produced by Alderson Research Laboratories, Stamford, CT) and the Radiological Physics Center (RPC) phantom,^(3)^ both of which provide an anatomically realistic external contour of a typical patient. The Rando phantom is constructed from a natural human skeleton cast inside material that is radiologically equivalent to soft tissue. The slab structure allows for the use of transverse films and a 3D array of thermoluminescent dosimeters (TLDs) for dosimetric verification. The RPC phantom consists of an anthropomorphic Perspex cast, inside which a cubic dosimetry insert able to hold TLDs and film can be positioned; the surrounding volume is then filled with water. Neither of these phantoms allows for the insertion of ionization chambers. The HANK phantom described in this work provides increased flexibility over other phantoms in terms of the available number and position of measurement points with either ionization chamber or film. The design of the phantom also means that it can be easily modified to incorporate inserts for other dosimeters such as microMOSFETs (metal oxide semiconductor field effect transistors) and fiberoptic detectors.

### A. Conventional/3D‐conformal plan tests

We used the HANK phantom to carry out an audit on the general method used for treatment planning at our institution. A PinPoint chamber (0.015 cm^3^) was used for all measurements. Low dose gradient measurements were repeated with a thimble ionization chamber (0.125 cm^3^). The ionization chambers were calibrated against a Farmer‐type chamber (30002: PTW), which in turn was traceable to the U.K. national standard. Plans were calculated on the Pinnacle^3^ TPS (version 7.4f), which incorporates a collapsed cone convolution dose algorithm, and were delivered on an Elekta Precise (Elekta, Crawley, UK) linear accelerator (LINAC).

To assess the reproducibility of measurement position achievable with the phantom, we carried out 3 investigations to establish a positional uncertainty:

- A PinPoint chamber was placed within the phantom at the edge of a field. Baseline reproducibility of delivery and measurement was established by 6 identical repeats without moving any component of the phantom or LINAC.
- To determine the repeatability of chamber positioning, the same field was delivered 6 times, each time removing and replacing the chamber.
- To ascertain positional uncertainty in the phantom setup, a field was delivered 6 times, resetting the phantom in 1 cardinal couch direction to the original position. This test was carried out in all 3 orthogonal couch directions, and the field was chosen so that the chamber was in jaw penumbra in the relevant direction.

The standard deviation (SD) in the dose measurement was related to a positional uncertainty by determining the dose gradient for each point from the TPS. The uncertainty was calculated as ±2 SD so as to give a 95% confidence level. The coefficient of variation (CoV) was also calculated (CoV=SD/mean).

**Figure 5:**
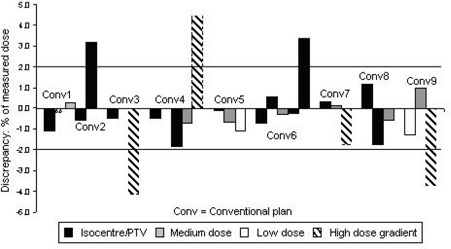
Results for simple treatments delivered to phantom, required to 2% accuracy. For all plans, isocenter dose was 200 cGy. All high dose gradient measurement doses were within 2 mm of the point dose within the planning system. Doses: isocenter /PTV (planning target volume)=180–220 cGy; medium=110–160 cGy;low=5–40 cGy. Conventional plans: Conv1=homogeneous superiorly− oblique wedged pair;; Conv2=homogeneous orthogonal wedged pair; Conv3=parallel opposed pair (isocenter only); Conv4=single anterior field; Conv5=three−field brick (pituitary); Conv6=heterogeneous superiorly−oblique wedged pair; Conv7=heterogeneous orthogonal wedged pair; Conv8=heterogeneous conformal nasal cavity; Conv9=heterogeneous wedged pair (orbit).

To verify the validity of using the phantom for all types of head‐and‐neck treatment, 9 other standard conventional treatments, listed in Fig. 5, were delivered to the phantom, including heterogeneous plans and conformal treatments. The treatments were delivered and assessed in order of increasing complexity so that the cause of any significant error could be isolated. The range of measurements performed was designed to establish whether the phantom would be capable of successfully auditing an IMRT program for head‐and‐neck patients. The measurement points depended on the particular plan and were chosen to be in high‐ and mid‐level dose regions and regions of sharp dose gradient.

The acceptability criteria of 2% or 2 mm set out by the International Commission on Radiation Units and Measurements were used to assess the suitability of the phantom for all non‐IMRT treatments.^(4)^ The 2‐mm acceptance dose range was determined by using points of interest in the planned dose distribution to calculate the maximum dose range 2 mm in each cardinal direction.

### B. IMRT plan tests

Head‐and‐neck IMRT was recently clinically implemented at the Christie Hospital. The procedure to verify the absolute dose is outlined here to highlight the utility of the HANK phantom. All cases were planned in line with the PARSPORT Trial protocol, using 5 beams at 6 MV (62−80 segments in total) to deliver 65 Gy to the primary PTV and 54 Gy to the nodal PTV in 30 fractions.^(5)^

**Figure 6:**
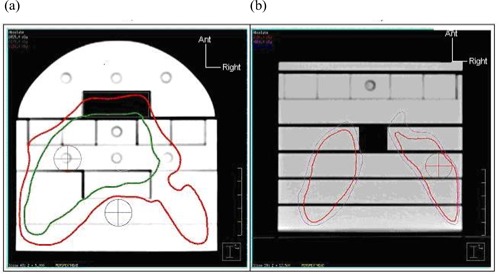
Computed tomography scan of phantom in the treatment planning system, illustrating (a) head section with simulated oral cavity and typical dose distribution with clinically relevant primary planning target volume (PTV, red) and spinal cord (blue) points of interest, and (b) shoulder section with nodal PTV measurement point (red) and trachea insert. Ant=anterior.

A computed tomography scan of the phantom (containing heterogeneities to represent the oral cavity and the trachea) was imported into the TPS. The clinical patient plan was transferred to the phantom, and the dose distribution was calculated. A sample of absolute dose values was taken from the 156 available measurement points (coordinates noted in the departmental protocol), chosen from regions of clinical or dosimetric interest within the dose distribution—that is, in the primary PTV, the nodal PTV, and the spinal cord [highlighted in Fig. 6(a,b)]. The points chosen were in stable areas of the combined dose distribution, avoiding steep dose gradients, so that the verification results were less likely to be erroneous as a result of small setup errors. Such a selection may not always be possible, particularly with a dose point representative of the spinal cord region, and so a range of potentially suitable points should be evaluated to choose one that is least affected by penumbrae.

The plan was delivered to the phantom, and a calibrated PinPoint chamber was used to take measurements at the chosen points. Overall dose action levels were chosen as 3% for the primary PTV and spinal cord, and 4% for the nodal PTV. The action level for individual beam errors was set at 5%.

Verification of the combined dose distribution was determined from the combination of all treatment beams involved in the delivery of a full fraction of a treatment to radiographic film positioned within the phantom. Transverse slices were calculated in the TPS as described earlier, using the phantom shown in Fig. 4, in which bisected films (resealed in a dark room) were then acquired 15 mm superior and 15 mm inferior to the isocenter for 3 patients. Coronal films were acquired within the main phantom for 3 patients.

## III. RESULTS

### A. Conventional/3D‐conformal plan tests

Table 2 shows the results of the repeatability of measurement and reproducibility of phantom setup; the findings demonstrate that the phantom can be set up and the dose to a point measured with a spatial precision of approximately ±0.5 mm. The chamber can be positioned to ±0.3 mm precision. Those results were calculated from CoVs of 0.005 and approximately 0.05 for chamber positioning and phantom setup respectively, values that compare to a CoV of 0.001 for repeated measurements without moving the phantom, chamber, or LINAC components. These findings demonstrate that the phantom setup has the precision required to measure dose in sharp field gradients, an attribute essential for IMRT commissioning.

Fig. 5 shows the results for absolute dose verification for all measured points; the findings indicate good agreement for most points (measurements to within ±2% of TPS prediction). The errors exceeding tolerance were either in high dose gradients or measured at beam penumbrae that entered the phantom through a sharp change in shape. The TPS is known not to model some sharp changes in shape well, because there is no requirement in clinical practice to model them. However, for a single‐field treatment, the TPS did model a beam with the central axis passing through a sharp change in phantom shape to within 2% and 2 mm of the measured dose. It can therefore be concluded that the problem lies in modeling sharp changes in phantom shape within the beam penumbra. Measurement points were subsequently chosen to avoid measurements within the beam penumbrae that passed through such changes in shape. This problem was not considered significant with regard to the clinical use of the TPS, because such changes in shape are not encountered clinically.

**Table 2:** Reproducibility results^a^

*Uncertainty*	*Coefficient of variation* (±)	*Positional uncertainty* (+mm)
Chamber repositioning	0.004	0.03
Longitudinal setup	0.051	0.30
Vertical setup	0.031	0.27
Lateral setup	0.029	0.27
Root mean square setup uncertainty		0.49

a Positional uncertainty was determined from the treatment planning system, assuming a linear gradient from the measurement point to 2 mm from the point. Approximately 65 cGy was delivered from 200 MU in the center of the phantom.

### B. IMRT plan tests

**Figure 7:**
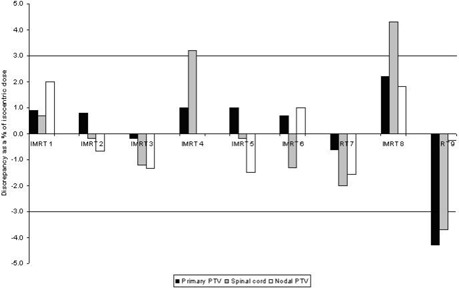
Results for absolute dose verification of clinically and dosimetrically relevant points for a sample of head‐and‐neck intensity‐modulated radiation therapy plans. PTV=planning target volume.

Fig. 7 shows the absolute dose verification results for 9 patient plans. The results for individual dose points have a mean error on the calculated dose to each point of +0.2% (range: −4.3% to +2.2%,n=9) for the primary PTV, −0.1% (range: −1.5% to +2.0%,n=8) for the nodal PTV, and 0.0% (range: −1.8% to +4.3%,n=9) for the spinal cord, indicating no systematic errors in measurement.

The measured differences for individual beams were found to exceed the 5% action level for, on average, 3 of the 15 measurements for each plan (3 measurements for each of 5 beams). In particular, discrepancies were found for measurements of the spinal cord point, an expected finding because of the high proportion of beam penumbrae that make up the dose in this region, making that point particularly sensitive to setup error or subtle errors in beam modeling. Tolerance was also exceeded for individual beams for measurements within the primary PTV.

**Figure 8:**
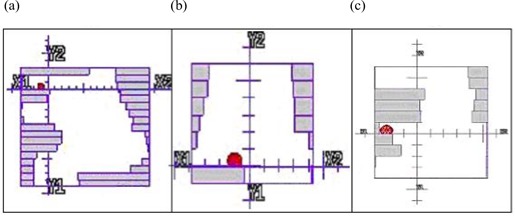
Illustration of segments causing measurement error in (a,b) primary planning target volume (PTV) point from the left anterior oblique beam, and (c) spinal cord point from the right anterior oblique beam. The red spheres represent the measurement points.

We investigated these discrepancies further by determining the error on individual segments and found them to correlate closely with heavily‐weighted segments in which the measurement point was found to be in or near the field penumbra and therefore susceptible to setup or delivery error, and also to correlate with subtle errors in the modeling of the beam penumbra by the TPS. Fig. 8 shows examples of these segments for the IMRT 9 plan.

These results suggest that the phantom and the beam model are both suitable for IMRT planning and verification but that, because of the inability of point measurement techniques to determine distance‐to‐agreement, plan verification must include methods to measure the dose distribution. Coronal films were acquired for 3 clinical plans between slabs 3 and 4 of the head section (extending to between slabs C and D of the shoulder section), corresponding approximately to a plane 1−2 cm anterior to the spinal cord, in which the dose distribution is considerably modulated in the attempt to minimize dose to the spinal cord. Gamma analysis showed good agreement with calculation, with a mean pixel failure of 1.6% (range: 0.5%−3.6%) within the 50% isodose at 4% and 4 mm.

**Figure 9:**
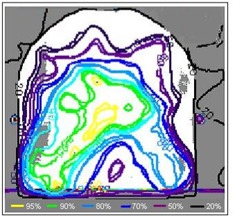
Typical gamma evaluation map at 5% and 5 mm for an axial slice at approximately the level of the parotids.

Fig. 9 shows a typical transverse gamma map for a combined treatment and demonstrates reasonably close agreement between calculation and measurement, although not as close as that seen with the coronal films. Results showed a mean pixel failure of 5.1% (range: 2.3%−9.4%) within the 50% isodose at 4% and 4 mm. The image does not appear to suffer from any artifacts as a result of cutting the film, but it does show areas of poor agreement between calculation and measurement toward the phantom surface, perhaps because of the known breakdown of the TPS dose calculation accuracy in that region.^(6)^

## IV. DISCUSSION

Our investigations into setup reproducibility and verification accuracy have provided confidence that the phantom developed in‐house is suitable for audit of conventional treatments with and without the use of multileaf collimators, and with homogeneous and heterogeneous plans. They have also served to audit the entire patient pathway for simple head‐and‐neck treatments from computed tomography scan through TPS to delivery. Our findings provided the necessary foundation to confidently use the phantom for IMRT audit purposes.

The HANK phantom has enabled systematic audit of the radiotherapy chain and development of IMRT verification procedures. It has highlighted a penumbra measurement problem inherent to the verification of IMRT plans for complex sites such as the head and neck, and as a result, absolute dose measurement points are, where possible, chosen in regions of low dose gradient so as to reduce the potential impact of setup errors in the measurement. Problems can arise from choosing measurement points in apparently stable areas within the full treatment plan dose distribution. Care must be taken when selecting measurement points to avoid positioning them in or near the penumbrae for several segments.

The additional measurement of the relative dose distribution with film is essential because of the lack of spatial information provided by point measurements of absolute dose using this phantom. The additional phantom shown in Fig. 4 can be used to acquire transverse slices through the head, although the accuracy of the resulting films appears limited as compared with the coronal films.

## V. CONCLUSION

A semi‐anatomic Perspex phantom was developed and is now in routine use for the verification of IMRT for the head and neck. The phantom incorporates heterogeneities to represent the oral cavity and trachea and can be used with ion chambers or film to measure dose points or dose distributions. It has a non‐uniform array of 156 possible measurement positions, from among which points of clinical interest can be chosen. The phantom can also be used to measure coronal and transverse dose distributions with radiographic film.

This simple phantom provides the accuracy and flexibility required for both IMRT technique development in the head and neck and for a thorough pre‐treatment verification program.

## ACKNOWLEDGMENTS

The authors acknowledge the early work carried out on the HANK phantom by J. Fairfoul, as well as the manufacture of the phantom and subsequent modifications carried out by the NWMP Workshop staff at the Christie Hospital.

## Supporting information

Supplementary Material
