# Supplementary material for: Design and implementation of a head‐and‐neck phantom for system audit and verification of intensity‐modulated radiation therapy
Source: J Appl Clin Med Phys. 2008 Apr 16;9(2):46–56. doi: 10.1120/jacmp.v9i2.2740 (PMC5721704; doi:10.1120/jacmp.v9i2.2740)
Supplement: Supplementary file 1 — Supplementary Material [file ACM2-9-046-s001.doc]

**Acknowledgements:**

The authors would like to acknowledge the early work carried out on the HANK phantom by J Fairfoul, as well as the manufacture of the phantom and subsequent modifications done by the Workshop staff at the Christie Hospital.
